# Cultural traditions across a migratory network shape the genetic structure of southern right whales around Australia and New Zealand

**DOI:** 10.1038/srep16182

**Published:** 2015-11-09

**Authors:** E. L. Carroll, C. S. Baker, M. Watson, R. Alderman, J. Bannister, O. E. Gaggiotti, D. R. Gröcke, N. Patenaude, R. Harcourt

**Affiliations:** 1 Scottish Oceans Institute, University of St Andrews, St Andrews, Fife, KY16 8LB, Scotland; 2 Department of Biological Sciences, Macquarie University, Sydney, NSW 2109, Australia; 3 School of Biological Sciences, University of Auckland, Auckland 1010, New Zealand; 4 Marine Mammal Institute and Department of Fisheries and Wildlife, Hatfield Marine Science Center, Oregon State University, Newport, OR 97365, USA; 5 Department of the Environment, Land, Water and Planning, Barwon South West Region, Warrnambool, VIC 3280, Australia; 6 Department of Primary Industries, Parks, Water and Environment, Hobart, TAS 7000, Australia; 7 The Western Australian Museum, Locked Bag 49, Welshpool DC, WA 6986, Australia; 8 Department of Earth Sciences, Durham University, Durham, DH1 3LE, United Kingdom; 9 Collégial International Sainte-Anne, Montréal, Québec, QC H8S 2M8, Canada

## Abstract

Fidelity to migratory destinations is an important driver of connectivity in marine and avian species. Here we assess the role of maternally directed learning of migratory habitats, or migratory culture, on the population structure of the endangered Australian and New Zealand southern right whale. Using DNA profiles, comprising mitochondrial DNA (mtDNA) haplotypes (500 bp), microsatellite genotypes (17 loci) and sex from 128 individually-identified whales, we find significant differentiation among winter calving grounds based on both mtDNA haplotype (F_ST_ = 0.048, Φ_ST_ = 0.109, p < 0.01) and microsatellite allele frequencies (F_ST_ = 0.008, p < 0.01), consistent with long-term fidelity to calving areas. However, most genetic comparisons of calving grounds and migratory corridors were not significant, supporting the idea that whales from different calving grounds mix in migratory corridors. Furthermore, we find a significant relationship between δ^13^C stable isotope profiles of 66 Australian southern right whales, a proxy for feeding ground location, and both mtDNA haplotypes and kinship inferred from microsatellite-based estimators of relatedness. This indicates migratory culture may influence genetic structure on feeding grounds. This fidelity to migratory destinations is likely to influence population recovery, as long-term estimates of historical abundance derived from estimates of genetic diversity indicate the South Pacific calving grounds remain at <10% of pre-whaling abundance.

Fidelity to migratory routes or destinations is a common trait across many taxa, including fish^1^, lizards^2^, birds and mammals^3^, both terrestrial e.g. ungulates^4^ and bears^5^ and marine e.g. cetaceans^6^ and pinnipeds^7^. Natal fidelity is one of the most well-studied exemplars, and particularly influences population structure and connectivity in marine species as diverse as leatherback turtles *Dermochelys coriacea*^8^, bull sharks *Carcharhinus leucas*^9^, and humpback whales *Megaptera novaeangliae*^10^.

Migratory traditions between two sets of habitat patches, breeding and non-breeding (typically feeding), are often a driver of population structure across an entire migratory network^11^. The effect is likely to be strongest when there is parental influence on migratory destinations. In species with long periods of parental care there is the opportunity to transmit parental preferences for breeding or feeding grounds to offspring^12^, a process termed migratory culture. If young animals show fidelity to natal breeding grounds and parental feeding grounds, the impact on population structure is likely to be strong. Alternatively, dispersal from or gene flow between natal breeding and parental feeding grounds might be sufficient to negate the effect of migratory culture on population structure. Even a small proportion of individuals deviating from the norm of migratory culture can represent a level of gene flow that can reduce genetic differentiation between populations^13^. However, few studies have been able to assess the effect of migratory culture on connectivity and genetic structure across a migratory network.

Southern right whales (*Eubalaena australis*) have migratory networks spanning thousands of kilometres, from sheltered coastal wintering grounds to offshore summer feeding grounds. The species was the target of a prolonged hunt across the southern hemisphere, with up to 150,000 southern right whales killed between 1790 and 1980^14^. Around New Zealand and east Australia, it is estimated that historical whaling killed over 58,000 southern right whales^15^. Coastal and pelagic fisheries also killed an unknown, but likely substantial, number of whales off the southern coast of South and Western Australia^16^,^17^. There was no discontinuity in the inferred historical distribution along the coast of Australia to indicate differentiation of areas into different management units^18^. A large-scale migration pattern, termed the counter-clockwise pattern (Fig. 1), has been inferred from historical whaling records. This suggests whales migrated in an east to west pattern from Tasmania to Western Australia, thereby linking whales across the southern coast of Australia^19^. There was also a suggestion that whales migrating up the west coast of Tasmania may head north and then out into the Tasman Sea; perhaps mixing with right whales wintering in New Zealand waters^19^.

Today, however, there is an apparent difference in the population size and rate of recovery of the species on its main Australian wintering grounds. Wintering grounds in Western and South Australia (southwest Australia), including key calving grounds, show a high degree of interchange based on the movement of photo-identified individuals^20^. Southwest Australia shows a strong rate of population growth and was estimated to number 2900 whales in 2010^21^. In contrast, the only area where whales are seen regularly in the southeast is Warrnambool, Victoria, with small numbers of whales seen each year in other parts of Victoria, Tasmania, and New South Wales^22^. Photo-identification studies suggest there is a degree of interchange within these areas^22^, collectively termed southeast Australia. Aerial surveys, combined with modelling of whale surfacing probabilities, produced an estimate of 257 whales in southeast Australia in 2014^22^.

The delineation of southeast and southwest Australia into distinct management units was proposed based on the difference in recovery rate and photo-identification data suggesting movement within each region is greater than between the two regions^20^,^22^. This hypothesis was previously tested using genetic data that showed southeast Australia, represented by Victoria and New South Wales, and southwest Australia management units were genetically differentiated from each other and from the New Zealand wintering ground, based on mitochondrial control region (mtDNA) haplotype frequencies^23^.

This finding, although preliminary as it was based on a small sample size from southeast Australia (*n* = 13), is consistent with the hypothesis of maternally directed fidelity to wintering grounds. Female, and to a lesser extent male, southern right whales show long-term fidelity to their natal wintering ground, based on long-term photo-identification and paternity studies^20^,^24^,^25^,^26^. It is hypothesised that such cultural traditions could lead to differences in mitochondrial DNA (mtDNA) haplotype frequencies between wintering grounds, as is seen across the South Pacific^23^,^27^ and indeed on a global scale^28^.

There is also limited evidence for maternally directed fidelity to summer feeding grounds. Valenzuela *et al.*^12^ used carbon and nitrogen stable isotope profiles to infer the foraging grounds of females sampled on the Península Valdés wintering ground. They report an association between the genetics and stable isotope data, such that whales with the same mtDNA haplotype were more likely to have similar stable isotope profiles than whales with different mtDNA haplotypes^12^. The most parsimonious explanation for this finding is maternally directed fidelity to feeding grounds leading to groups of loosely related individuals foraging in the same feeding grounds.

Overall, cultural traditions to migratory destinations could potentially both promote and/or reduce genetic differentiation across a network of winter breeding/calving grounds and summer feeding grounds in the southern right whale. Female fidelity to migratory destinations has led to structuring of maternally inherited mtDNA haplotypes on both winter calving and summer feeding grounds. If such cultural traditions are also preserved by males, it could lead to similar structuring of bi-parentally inherited DNA markers. Much depends on when and where mating occurs. If mating occurs on or en route to feeding grounds and whales from distinct wintering grounds mix on shared feeding grounds, this could decrease genetic differentiation. For example, stable isotope analyses and satellite tagging studies suggests that whales from the Argentinean and South African wintering grounds may mix and potentially mate on shared feeding grounds^29^,^30^. However, if mating occurs on wintering grounds, to which males and females show fidelity, this could increase genetic differentiation. The only paternity analysis in the species to date suggests that males from the New Zealand wintering ground show fidelity to this region and father New Zealand calves^24^, which could increase the degree of differentiation between wintering grounds.

On the Australian wintering grounds, there is a third possibility: that whales from the southeast and southwest wintering grounds mix on coastal migratory corridors along the coast of Australia. This could occur under the counter-clockwise migration pattern: some whales migrating towards southwest Australia start their migration in southeast Australia, so whales from both management units are found in shared migratory corridors. This idea is supported by the finding that 95% of within-year resights of photo-identified whales show an east to west movement pattern across the Australian coast^20^. If mating occurred on shared coastal migratory corridors, then it would decrease the level of genetic differentiation between these management units.

Here we use a combination of genetic and stable isotope data to investigate the role of migratory culture in shaping the recovery and connectivity of the southern right whale in the South Pacific. We reassess the preliminary findings of genetic differentiation between the southwest and southeast Australian management units, utilising a larger sample, one both temporally and spatially enhanced, including analysis from new genetic markers and incorporating stable isotope profiles. Specifically, we use mtDNA and bi-parentally inherited microsatellite data to test the hypotheses that migratory fidelity in the southern right whales has led to genetic differentiation between (1) New Zealand and Australian calving grounds, (2) previously defined southwest and southeast Australian management units (pooled migratory corridor and calving ground samples), with New Zealand as a comparator, and (3) between migratory corridors and calving ground samples. In addition, we use a combination of genetic and stable isotope data to investigate the influence of migratory fidelity to feeding grounds on population structure by testing the hypotheses (1) there is maternally directed fidelity to feeding grounds in Australian southern right whales and (2) that there is a correlation between genetic estimates of relatedness and feeding ground preferences. Finally, we use mtDNA data to provide long-term estimates of genetic diversity and historical population size of the right whale populations in the South Pacific. In doing so, we provide an understanding of the role of migratory culture as a driver of genetic structure in the contemporary population and provide targets to inform our understanding of the recovery of the species in the New Zealand and Australia.

## Methods

Small samples of skin were collected from southern right whales across New Zealand and Australia using a stainless-steel biopsy dart fired from a modified veterinary capture rifle^31^ or deployed from a crossbow^32^. Samples were collected from Bremer Bay/Doubtful Island Bay, Western Australia in 1994 and 1995 as previously described^23^, and Cape Jervis/Encounter Bay, South Australia, Warrnambool and Port Fairy, Victoria, along the coast of Queensland, New South Wales, and south east Tasmania between 2001 and 2013. In New Zealand, samples were collected from southern right whales around the NZ sub-Antarctic (2006–2009) and mainland New Zealand (2003–2010) wintering grounds as previously described^24^ and a subset (*n* = 51) were used here as a comparison. Sampling was carried out following protocols that were approved by the Macquarie University Animal Ethics Committee, Department of Primary Industries Parks Water and Environment Animal Ethics Committee and the University of Auckland Animal Ethics Committee. Sampling was conducted in accordance with and under permits from: Department of Environment, (Australia); Department of Environment and Heritage (South Australia); Department of Primary Industries, Parks, Water and Environment (Tasmania); Department of Natural Resources and Environment (Victoria); Office of Environment and Heritage (New South Wales); Department of Conservation and Land Management (Western Australia) and the New Zealand Department of Conservation (New Zealand).

We broadly categorise sampling areas into calving/nursery areas (Western Australia, Warrnambool in Victoria and New Zealand) and migratory corridors (South Australia, Queensland, New South Wales, Port Fairy in Victoria and Tasmania). Calving grounds are areas used regularly by females with calves who show long-term fidelity to such sites^20^, and migratory corridors are coastal areas where whales are regularly seen but have comparatively shorter residency times^33^. We additionally separate samples into the previously described^23^ New Zealand, southeast and southwest Australian management units. Here we define management units as demographically independent subpopulations where recruitment from within the management unit is more important to its maintenance than immigration from neighbouring subpopulations^6^,^34^.

It should be noted that the majority of New Zealand samples come from the sub-Antarctic Auckland Islands, which is a key wintering ground for all demographic classes and so represents both a key calving area and management unit. Samples comprise those used in previous analyses^23^ and samples not previously analysed (Table 1, Supplementary Table 1). Samples were stored in 70% ethanol in the field and transferred to −20 °C storage at the University of Auckland until further analyses.

### DNA and stable isotope profiles

We constructed DNA profiles, comprising genetically identified sex, mtDNA haplotype (500 bp) and microsatellite genotype (up to 17 loci), for southern right whale samples collected around New Zealand and Australia. This was a mixture of previously published data and newly generated data (Table 1, Supplementary Material 1, Supplementary Table 1 and 2). In addition, we constructed stable isotope profiles (δ^13^C and δ^15^N) for southern right whales skin biopsy samples collected in Australia (Supplementary Material 1).

### Analysing wintering ground population structure

For the mtDNA data, Arlequin v3.5^35^ was used to estimate haplotype (*h*) and nucleotide (*π*) diversity for each sampling location and putative management unit. Differentiation between (1) Western Australian, Victorian and New Zealand calving grounds and (2) the southwest Australia, southeast Australia and NZ management units, was estimated using pairwise F-statistics (F_ST_), Ф_ST_ and a hierarchical analysis of molecular variance (AMOVA^36^,^37^), calculated in Arlequin v3.5. The significance of these differences was tested using a permutation procedure in Arlequin v3.5 (10,000 permutations, with significance set at α = 0.05). Given the small size of some of the samples, we also carried out comparisons using an exact test of differentiation (1,000,000 Markov chain steps; 1,000,000 dememorization steps, with significance set at α = 0.05).

For the microsatellite data, we estimated the mean number of alleles per locus and observed and expected heterozygosity for each sampling location and management unit using CERVUS v3.0^38^. Pairwise and overall F_ST_ values for microsatellite loci were calculated in GENEPOP v4.0^39^ and the exact *G* test was used in the same program to test for significant differences in allele frequencies between (1) calving grounds and (2) management units. We also estimated Jost’s D statistic^40^ to compare microsatellite allele frequencies between regions using GENODIVE v2.0b1^41^.

We also investigated the hypothesis that individuals from different calving grounds were mixing on the same migratory corridor. This was done by calculating pairwise mtDNA-based F_ST_ and Ф_ST_ and microsatellite-based F_ST_ and Jost’s D, between the calving grounds and migratory corridors, as described above (the Victorian migratory corridor was excluded due to a small sample size). We used Genetix^42^ to undertake a Factorial Correspondence Analysis (FCA)^43^ to visualise the relationships of calving grounds and calving grounds with migratory corridors.

### Testing for maternally directed fidelity to feeding grounds

The hypothesis of maternally directed fidelity to feeding grounds leads to the expectation that whales that share mtDNA haplotypes are more likely to have similar stable isotope profiles than those with different mtDNA haplotypes. We conducted several analyses to investigate the relationship of stable isotopes with both the mtDNA and microsatellite data, using the Australian samples only. First we tested the data for normality and plotted δ^13^C and δ^15^N by mtDNA haplotype to visually identify any patterns. We then tested for significant differences in δ^13^C and δ^15^N values between individuals with different mtDNA haplotypes using the Kruskal-Wallis test.

We used generalised linear modelling in R^44^ to investigate whether mtDNA haplotype, sex or sampling area (i.e. Australian state of migratory corridor or calving ground) explained any variation in δ^13^C or δ^15^N values. Model fit was assessed using AIC and estimated the size of the effect of mtDNA on stable isotope values by assessing the weight of models that included mtDNA as a explanatory factor^45^. In addition, we used the non-parametric randomisation test of Valenzuela *et al.*^12^ to test the hypothesis that under the assumption of maternally directed fidelity to feeding grounds, the isotopic difference between two whales from the same matrilines should be smaller, on average than the distance between two whales from different matrilines. The program implements a testing procedure in which the original dataset is randomised 10,000 times and the F-ratio (computed as the ratio of between- to within-group mean squares) is calculated for each randomised dataset. This procedure leads to a null-distribution of the F-ratio that is then used to calculate the significance of the observed F-ratio. More precisely, the significance is calculated as the proportion of randomly-generated F-values that are as extreme or more extreme than the observed value. Unlike Valenzuela *et al.*^12^, which only considered samples from a single wintering ground, we have samples from across a region that shows population structure. The most common haplotype represented 30% (24/66) of samples and a broad isotopic range. In order to account for the likelihood that this single mtDNA haplotype represents multiple matrilineal groups, we categorised individuals by management unit (southeast Australia versus southwest Australia).

To further investigate the relationship of the genetic and stable isotope data, we tested the hypothesis that individuals that show a higher level of relatedness, based on their microsatellite genotypes, have more similar isotopic profiles. To do this, we estimated the Euclidean distance between individuals based on their δ^13^C and δ^15^N values. Pairwise genetic similarity between individuals was calculated in the program COANCESTRY^46^, using the microsatellite-based relatedness estimators that work best in loosely-related groups of individuals: those of Ritland^47^ and Lynch & Ritland^48^. We undertook Mantel tests of the Euclidean distances of δ^13^C and δ^15^N values and the relatedness estimators using the R package ape^49^. Significance was assessed using 10,000 permutations.

### Estimating genetic diversity and historical population size using mtDNA haplotype sequences

We used program LAMARC^50^ to estimate the level of genetic diversity, θ, for each of the calving grounds, using the mtDNA haplotype sequence data. LAMARC was chosen because it avoids potential biases in the estimation of θ by simultaneously estimating migration rates, growth rates and θ. Thus, unlike other approaches, we avoid the upward biases in diversity estimates when migration is ignored and we also account for fluctuations in abundance in these populations caused by whaling. Three replicates of LAMARC were run, each with initial chains of 5,000 sampled genealogies, with 10,000 excluded as burn-in, and two final chains with 50,000 sampled genealogies, sampled every 50^th^ genealogy, discarding the first 20,000 of each search.

We converted θ into census population size (*N*_*C*_) following Roman and Palumbi’s^51^ use of the formula θ = 2 N_*e(f)*_μ, where μ is the substitution rate per generation and N_*e(f)*_ is effective female population size. We took 2,000 samples of θ from the LAMARC output (95% HPD intervals) and for each we estimated N_*e(f)*_ using: (1) the mutation rate of 2.0 ×10^−8^ bp^−1^ year^−152^, estimated from the closely-related bowhead whale *Balaena mysticetus* and previously used in similar analyses for right whales^14^ and (2) an estimate of generation span that represents the populations under a range of conditions, from current growth to pre-disturbance conditions, modelled as a uniform distribution (18.1–28.8 years)^53^. We converted the estimate of *N*_*e(f)*_ to *N*_*C*_ using two correction factors. Firstly, we adjust *N*_*e(f)*_ to number of mature females, *N*_*T(f)*_, by multiplying by two^51^. Secondly, we converted the *N*_*T(f)*_to *N*_*C*_ by multiplying *N*_*T(f)*_by a value sampled from a uniform distribution between 2.5 and 4.71. This distribution represents the ratio of the proportion of reproductively mature females in a population to the total population size, in pre-disturbance, equilibrium conditions (2.5: derived from the estimate of ~40% of the population will be mature females when population growth rate is 0^53^) to one undergoing maximal growth (4.71^54^). These 2,000 simulations were used to calculate mean and 95% CL for N_*e(f)*_ and *N*_*C*_. By accounting for uncertainty in generation span and proportion of mature females in the population, we attempt to account for fluctuations in the population size over time, due to whaling or other historical bottlenecks.

## Results

### Genotyping and levels of genetic diversity

There was variation in sample quality and quantity, so not all samples were genotyped at all 17 loci. However, a total of 86 Australian and 51 NZ samples were genotyped at between 10 and 17 loci (average 15.7 loci): see Table 1 for sample sizes per sampling location. No loci significantly deviated from the Hardy-Weinberg equilibrium and there was no evidence of significant linkage across the loci. Internal controls were amplified 2284 times and there were 14 single allele errors detected, giving an estimated per-allele error rate of 0.61%. Comparison of genotypes revealed several individuals had been resampled, based on an average of 11.5 matching loci providing P_ID_ of 2.84 × 10^−14^ (Supplementary Table 3). Only one copy of each unique genotype was retained per sampling location for further analyses.

**Figure 1:**
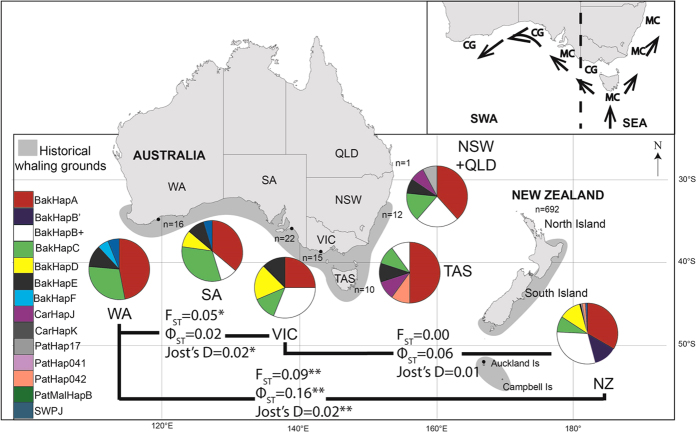
Main figure shows sample sizes and pie charts showing mitochondrial control region (mtDNA) haplotype frequencies for southern right whale calving grounds in Western Australia (WA), Victoria (VIC) and New Zealand (NZ) and migratory corridors in South Australia (SA), Tasmania (TAS) and New South Wales (NSW). The Queensland (QLD) is included in the NSW piechart and the VIC migratory corridor sample is not shown. The level of differentiation between calving grounds (WA, VIC and NZ) are shown by the mtDNA-based F_ST_ and Φ_ST_ and microsatellite-based Jost’s D. The inset shows the ‘counter-clockwise’ migration pathway of southern right whales along the coastal wintering grounds of Australia. Also shown is the position of the calving grounds (CG) and migratory corridors (MC), with the delineation into southwest Australia (SWA) and southeast Australia (SEA) management units shown with dotted line. Map was created in Adobe Illustrator. *p < 0.05, **p < 0.01.

There were between 5 and 6 mtDNA haplotypes per region, and each had reasonably high levels of haplotype diversity, with the exception of New Zealand as previously characterised (Supplementary Tables 4 and 5). BakHapA is the most common haplotype across all calving grounds and migratory corridors. All haplotypes were shared between at least 2 regions or management units, with the exception of BakHapF that was private to Western Australia and CarHapK and BakHapB’ that were found only in New Zealand (Fig. 1). Some haplotypes show regional patterns. For example haplotype SWPJ is unique to the Western Australian calving ground and the South Australian migratory corridor, whereas the New South Wales and Tasmanian migratory corridors share rare haplotypes with the New Zealand calving ground (CarHapK and PatHap04.2). However, the sample sizes are too small from the some locations to make definitive conclusions. In addition, the microsatellite loci also showed high levels of genetic diversity across regions and management units (Supplementary Table 4).

### Testing for population structure on wintering grounds

We tested for population structure on wintering grounds in two ways: testing first between calving grounds and then between management units, the latter comprise different habitat types including calving grounds and migratory corridors.

### Genetic differentiation between calving grounds

There was significant differentiation among calving grounds found in the microsatellite data (F_ST_ = 0.0086, p < 0.01) and mtDNA data, based on both the haplotype frequencies (F_ST_ = 0.048, p < 0.01) and haplotype distances (Φ_ST_ = 0.109, p < 0.01). There was a significant difference between New Zealand and Western Australia based on both mtDNA and microsatellite data (Table 2). There was also a significant difference between Victoria and Western Australia based on microsatellite loci and based on mtDNA F_ST_, but not mtDNA Φ_ST_. There was no significant difference between Victoria and New Zealand.

### Genetic differentiation between management units

There was significant differentiation among management units found using AMOVA in the mtDNA data, based on both haplotype frequencies and distances (F_ST_ = 0.044, Φ_ST_ = 0.096, p < 0.01), but not microsatellite data (F_ST_ = 0.0042, p = 0.15). As found previously, there was significant differentiation between southwest Australia and New Zealand based on both microsatellite allele and mtDNA haplotype frequencies. There was some differentiation between southeast Australia and New Zealand based on mtDNA data but not microsatellite allele frequencies (Table 2). No difference was found between the two putative Australian management units.

### Genetic differentiation between calving grounds and migratory corridors

There was no significant differentiation between the Australian calving grounds and migratory corridors, based on either mtDNA haplotype or microsatellite allele frequencies (Supplementary Table 6). The exception to this was the Tasmanian migratory corridor, which was significantly different to both the South Australian migratory corridor (F_ST_ = 0.010, Jost’s D = 0.036, p < 0.05) and the Western Australian calving ground (F_ST_ = 0.018, Jost’s D = 0.062, p < 0.01). There was a significant difference between the South Australia migratory corridor and the New Zealand calving ground (mtDNA-based F_ST_ = 0.055, p < 0.05, Φ_ST_ = 0.144, p < 0.01). However, New Zealand was not significantly different to either the Tasmanian or the New South Wales migratory corridors (at p < 0.05).

**Figure 2:**
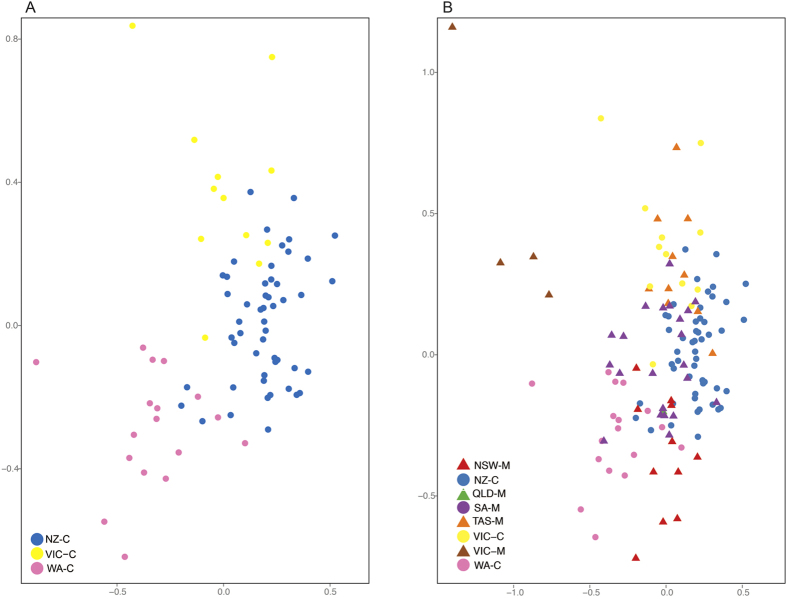
Factorial Correspondence Analysis results, based on 17 microsatellite loci, of (A) southern right whale calving grounds in New Zealand (NZ-C), Victoria (VIC-C), and Western Australia (WA-C) and (B) southern right whale calving grounds and migratory corridors in New South Wales (NSW-M), Tasmania (TAS-M) and South Australia (SA-M).

Inspection of the FCA results using the calving ground samples only show individuals from each calving ground group together, but with some overlap between calving grounds (Fig. 2). Further inspection of the FCA results from both calving grounds and migratory corridors shows that migratory corridor samples overlap the distribution of multiple calving grounds (e.g. South Australia overlaps with Victoria and Western Australia) except for the Victorian migratory corridor. The latter did not overlap with any calving ground samples.

### Testing for maternally directed fidelity to feeding grounds

The measurements of δ^13^C and δ^15^N for individual whales were normally distributed based on the Shapiro-Wilk normality test. We found a significant difference in δ^13^C values (Kruskal-Wallis p = 0.002 p = 0.015), but not δ^15^N values (p = 0.771 p = 0.760) associated with mtDNA haplotypes. Given the latter, we only report the comparison of genetic data with δ^13^C values (see Supplementary Material 2 for δ^15^N data).

**Figure 3:**
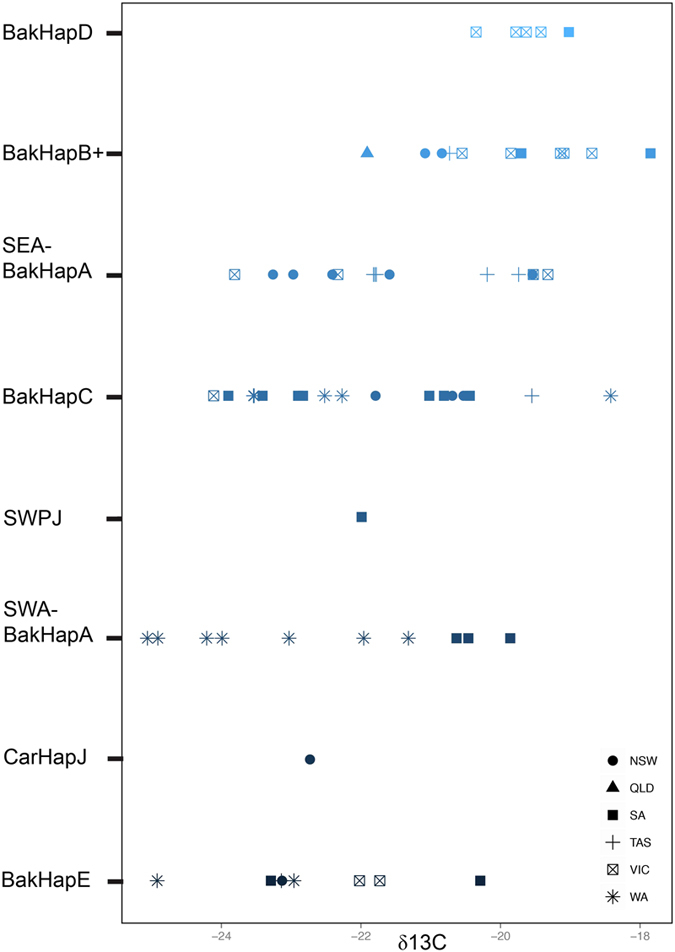
Scatterplot of δ^13^C values from southern right whales samples plotted by mitochondrial DNA control region (mtDNA) haplotype.

Plotting the mean of δ^13^C against two-way combinations of factors revealed interactions, so we considered general linear models with interaction terms. For the δ^13^C data, the best-supported model according to AIC indicated that mtDNA, sex and state were all influential in determining δ^13^C (Fig. 3). Model weighting supported the hypothesis that mtDNA is a strong factor on determining δ^13^C value, as > 95% of model weighting was given to those models that incorporated mtDNA haplotype as an explanatory factor (Supplementary Table 7).

The randomisation test of Valenzuela *et al.*^12^ revealed that the isotopic difference in δ^13^C (p = 0.004) between two whales from the same matrilines was on average smaller than the distance between two whales from different matrilines. There was also a significant relationship between the microsatellite-based relatedness estimates of Ritland^47^ (p = 0.003) and Lynch and Ritland^48^ (p = 0.005) and the Euclidean distances of δ^13^C, based on the results of the Mantel and permutation tests.

### Estimating genetic diversity and historical population size

We used LAMARC to estimate θ for each calving ground while accounting for the effect of migration and changes in population sizes. From these estimates we derive estimates of historical population size. Inspection of the LAMARC output in the program TRACER^55^ indicated the runs converged. Estimates of θ were derived from effective sample sizes of >1000 and are shown with derived estimates of *N*_*e(f)*_and *N*_*C*_ in Table 3 (migration rates are in Supplementary Table 8). The estimate of θ and *N*_*C*_were similar between New Zealand and Western Australia, with historical abundances of 30–35,000 whales. In contrast, θ and *N*_*C*_ for Victoria was lower with historical abundance estimated at 22,000 whales.

## Discussion

### Maternally directed fidelity of migratory destinations leads to population structure across a migratory network

Here we demonstrate genetic structure at both ends of a migratory network, winter calving grounds and summer feeding grounds, in the Australian southern right whale. This population structure appears to be driven by migratory culture, which is consistent with long-term behavioural studies that show migratory fidelity appears in general to persist across a lifetime in females (although there are exceptions^56^). On an evolutionary timescale, maternally directed fidelity to calving grounds appears to have driven significant differences in mtDNA haplotype frequencies between southern right whale calving areas across Australia, and indeed across the world^28^. We also found significant differences in microsatellite allele frequency data between calving grounds. This is consistent with some degree of fidelity by male southern right whales to wintering grounds, as previously suggested by photo-identification data and paternity analyses^20^,^24^,^30^.

Using a combination of genetic and stable isotope data, we also find evidence for maternally directed learning of summer feeding grounds from samples across Australia, with similarities to that found within the Argentinean southern right whale wintering ground^12^. The significant relationship between stable isotope profiles and both mtDNA haplotype and microsatellite-based estimators of relatedness indicates migratory culture to feeding grounds is likely to be a strong driver of genetic structure across migratory networks and is consistent with the idea that both sexes show some degree of fidelity to summering grounds.

Migratory culture in the southern right whale, and other baleen whales, likely represents a more successful strategy than random dispersal. This could be because fidelity to the maternal migration route has already proven to lead to reproductive success, while adopting a novel route has an unknown, and perhaps more risky, chance of success^13^. However, in a rapidly-changing environment, where previously productive feeding grounds could become sub-optimal in future, such migratory conservatism could reduce reproductive success. For example, reproductive success in southern right whales in Argentina has been linked to environmental conditions at one of the population’s feeding grounds^57^. Fidelity to a sub-optimal feeding ground could be leading to nutritional stress in females and contributing to a recent increase in juvenile mortality events at the Argentinean wintering ground^58^.

Our analyses also indicate on-going gene flow between calving grounds and, more broadly, between previously defined management units. While there was significant differentiation between the Western Australian and other calving grounds, there was no differentiation between the Victoria and New Zealand calving grounds. This was somewhat surprising given previous findings of some difference in mtDNA haplotype frequencies^23^, a lack of direct matches between the two regions, based on the comparison of either photo-identification data or DNA profiles, and the difference in the recovery rate between the two regions. The New Zealand population was estimated to number 2,139 whales in 2009 and to be growing at 7% per annum, based on a capture-recapture study^59^. The population appears to be recolonizing former winter habitat around mainland New Zealand^60^, so it is possible that some whales from the New Zealand population are also migrating to southeast Australia. Additionally, there is a suggestion from historical data that whales from the two regions could have mixed in the Tasman Sea, facilitating gene flow.

### Fine-scale population structure driven by differences in habitat use

While there is significant difference between the calving grounds, the picture becomes more complicated when considering the relationship of calving grounds and migratory corridors. There was evidence for mixing of whales from discrete calving grounds on shared migratory corridors. For example, the South Australia and New South Wales migratory corridors were not significantly different from either of the adjacent Victorian or Western Australian calving grounds. The FCA results also support the hypothesis that whales from distinct calving grounds are mixing on the wintering grounds. However, there was a limit to this mixing: the Tasmanian migratory corridor was genetically differentiated from the Western Australian calving ground based on microsatellite allele frequencies and the New Zealand calving ground was genetically distinct from the South Australian migratory corridor based on mtDNA haplotype data.

This mixing of whales from distinct calving grounds on shared migratory corridors is also consistent with the ‘counter-clockwise’ migration hypothesis^20^. This hypothesis posits that whales from calving grounds across Australia mix in migratory corridors in southeast Australia, before those travelling to the more westerly calving grounds continue to Head of Bight, South Australia or Western Australia. It also provides a mechanism for gene flow between calving grounds in Australia, assuming mating occurs on the wintering grounds.

If present, such migration patterns could lead to a decrease in the signature of genetic differentiation between the two Australian management units when migratory and calving area samples are pooled. This may have contributed to the decrease in the estimate of differentiation between the two putative Australian management units in this study compared with previous research (F_ST_ = 0.15, Φ_ST_ = 0.12, p < 0.001^23^) and is in contrast with the comparison of the pure calving ground samples. However, this may also be a sampling effect, as earlier findings were based on a small sample size from southeast Australia (*n* = 13). The inclusion of whales ‘from’ southwest Australian calving grounds in the southeast Australian management unit via migratory corridor samples could contribute to the increase in differentiation seen between the southeast Australian and New Zealand management units, compared with the calving ground samples.

### Genetic diversity and historical abundance

The estimates of historical abundance range from 22,000 whales for the Victoria calving ground, representative of the southeast Australia management unit, to 35,000 for the New Zealand calving ground, the latter representative of the overall New Zealand management unit. Migratory corridors were not included, as they could comprise individuals from multiple calving grounds, confounding these analyses. The long-term estimate for New Zealand is very similar to the estimate of pre-whaling abundance of 27,000 whales (22,000–32,000 whales), derived from a logistic growth model incorporating a regional catch series and constrained by genetic data^61^. Our estimates are also consistent with those from a similar θ-based approach, which estimated the long-term circumpolar southern right whale population size to be 202,000–370,500 for southern right whales^14^.

Global estimates of pre-whaling abundance, estimated using logistic growth modelling and a global catch series, had previously suggested that the historical circumpolar population size was substantially smaller, at 55,000–100,000 whales^62^. There is a potential temporal mismatch between the two estimates: the catch-series method estimates the global population size in 1770, whereas the genetic estimate is a long-term mean rather than an estimate for a specific time point. However, the catch series estimates did not account for population structure and uses an incomplete catch series that started in 1770, despite our knowledge that whaling began as early as 1603 in some regions^62^. Therefore the catch series method may be estimating the abundance of an already-depleted global population. Genetic-based estimates of long-term population size have many caveats^63^, and though we attempted to account for uncertainty in the demography over time we acknowledge that there will be unaccounted uncertainty in the estimates caused by using a fixed mutation rate. Exploring a range of mutation rates did not substantively change the point estimates of the historical abundance estimates but did increase the 95% CL (Supplementary Table 9). Finally, we acknowledge these estimates are based on one locus and have broad confidence limits.

### Limitations and potential biases

The low abundance and wide range of the previously heavily exploited southern right whale off southeast Australia meant that the sample size used here (*n* = 39) took 20 years to collect. The dataset therefore contains samples from whales of different ages, sex, reproductive state and migratory status, all of which may also influence the stable isotope profile^64^. For example, relying on blubber to maintain metabolism during migration or lactation will mean an animal has a lower δ^13^C than when it is not relying on fat stores^64^. Concordant with the latter, we found an effect of sex on the stable isotope profile from generalised linear model ranking. However, the effect of mtDNA haplotype on δ^13^C is moderately strong, suggesting that maternally directed fidelity to feeding grounds is very likely and overrides other factors. We suggest that the implications of this finding may be important in other stable isotope analysis studies. Stable isotope analyses are often conducted in the absence of concurrent genetic analyses, and for rare and uncommon animals frequently opportunistic rather than truly random or systematic. These findings suggest caution in both sampling design and in interpretation when underlying drivers such as those found here cannot be attributed.

That δ^15^N was not significantly associated with mtDNA may indicate the whales were feeding at similar trophic levels throughout their range and is not unexpected^65^. Prey items include calanoid copepods and krill^66^,^67^,^68^ that occupy similar trophic levels δ^15^N values^69^, perhaps leading to less differentiation in δ^15^N values between matrilines. Southern right whales that winter in New Zealand and Australia are thought to forage across a broad geographic range from the sub-tropical convergence to south of the polar front, Antarctica^70^,^71^. This naturally creates a broad potential range of δ^13^C values within a population from which to sample.

## Conclusion

Here we use a combination of genetic and stable isotope data to provide evidence for population structure across a migratory network, likely driven by fidelity to migratory destinations. This type of migratory culture has been inferred to explain the spatially variable recovery of baleen whales^6^, including the southern right whale^23^ and humpback whale^10^. When whales that show fidelity to a particular migratory destination are extirpated, the ‘memory’ of that migratory destination is also lost. The effect is exacerbated when there is depletion across the migratory network, as was the case with whaling^6^. This extirpation is likely contributing to the slow recovery of southern right whales in southeast Australia and mainland New Zealand^60^. Migratory culture to feeding grounds could also limit recovery if individuals show fidelity to sub-optimal feeding grounds. This is supported by the finding that reproductive success in the South Atlantic southern right whale has been linked to environmental conditions at feeding grounds^57^. However, the evidence of some genetic mixing identified here suggests that even with limited plasticity in migratory fidelity there is a likelihood that previously inhabited migratory destinations will be recolonized as the species recovers from whaling^60^,^72^.

## Additional Information

**How to cite this article**: Carroll, E. L. *et al.* Cultural traditions across a migratory network shape the genetic structure of southern right whales around Australia and New Zealand. *Sci. Rep.* **5**, 16182; doi: 10.1038/srep16182 (2015).

## Supplementary Material

Supplementary Information

## Figures and Tables

**Table 1:** Number of skin samples collected from southern right whales on calving grounds and migratory areas around New Zealand and Australia.

Region	Habitat	N_samples_	N_G_/N_N_	N_SI_	N_mtDNA_	M	F
QLD	Migratory	1	1/1	1	1	0	1
NSW	Migratory	16	12/8	11	12	3	9
TAS	Migratory	10	10/10	9	10	6	4
VIC	Calving	16	12/3	11	12	2	10
VIC	Migratory	5	4/4	3	4	2	2
SEA	MU	48	39/26	35	39	13	26
SA	Migratory	24	22/1	16	22	11	11
WA^A^	Calving	22	17/4	15	16	10	5
SWA	MU	46	39/5	31	38	21	16
AUS		94	78/31	66	77	34	40
NZ^B^	Calving/MU	51	51/0	0	692		

The number of total unique genotypes that passed quality control (N_G_), assumed to represent unique individuals after the removal of replicates, is listed, in addition to the number of genotypes new (not previously used in Carroll *et al.*^23^: N_N_). Also listed are the numbers of mitochondrial control region haplotypes (N_mtDNA_), stable isotope profiles (N_SI_), males and females associated with a unique individual sampled from each location. Sampling location abbreviations are New South Wales (NSW), Tasmania (TAS), Victoria (VIC), which are pooled to form Southeast Australia (SEA), and South Australia (SA) and Western Australia (WA), which are pooled to form southwest Australia (SWA), and New Zealand management units (NZ). SEA and SEA are pooled for the Australian (AUS) total. Sampling areas are defined as calving grounds, which are used regularly by females who show long-term fidelity to such sites, migratory corridors, coastal areas where whales are regularly seen but have comparatively shorter residency times, and management units (MUs), which are considered demographically isolated management units.

^A^2 samples from WA are missing sex ID info.

^B^The mtDNA data come from Carroll *et al.*^59^.

**Table 2:** Genetic differentiation of A. southern right whale calving grounds, based on samples collected from New Zealand (NZ), Victoria (VIC) and Western Australia (WA) and B.

A.	Microsatellites			mtDNA		
	NZ	VIC	WA	NZ	VIC	WA
2N/N	102	26	34	692	12	15
NZ		0.000	0.051**		0.028	0.164**
VIC	0.000		0.038*	0.000		0.057
WA	0.016**	0.011*		0.088**	0.098*	
B.	NZ	SEA	SWA	NZ	SEA	SWA
2N/N	102	78	78	692	39	38
NZ		0.010	0.025**		0.032*	0.158**
SEA	0.003		0.007	0.014*		0.033
SWA	0.007**	0.002		0.075**	0.015	

Management units, based on samples collected from New Zealand (NZ), southeast Australia (SEA) and southwest Australia (SWA). On the left-hand side of the table is the microsatellite-based F_ST_ (bottom left quadrant) and Jost’s D (top right quadrant) and on the right-hand side of the table are mtDNA-based F_ST_ (bottom left quadrant) and Φ_ST_ (top right quadrant). *p < 0.05; **p < 0.01.

**Table 3:** Estimates and 95% highest probability density (HPD) intervals of θ for southern right whale calving grounds in Western Australia (WA), Victoria (VIC) and New Zealand (NZ) and the derived statistics of mean and 95% confidence limits (95% CL) of effective female population size (*N*_*e(f)*_) and mean census population size (*N*_*C*_).

	WA	VIC	NZ
θ	4.72E-3	3.36E-3	5.55E-3
(95% HPD Interval)	(8.31E-5, 0.0115)	(5.41E-5, 9.15E-3)	(3.61E-4, 0.0138)
*N*_*e(f)*_	4,359	3,002	5,975
(95% CL)	(823, 11,140)	(408, 8,372)	(947, 12,849)
*N*_*C*_	31,150	21,697	35,460
(95% CL)	(5,324, 83,092)	(2,931, 63,435)	(6,674, 93,072)
Current abundance	2,900	257	2,300
(Year)	(2010^25^)	(2014^26^)	(2009^69^)

θ was estimated using mtDNA control region haplotypes (500 bp) and program LAMARC.
