# Corrigendum: Cultural traditions across a migratory network shape the genetic structure of southern right whales around Australia and New Zealand

**DOI:** 10.1038/srep21875

**Published:** 2016-03-04

**Authors:** E. L. Carroll, C. S. Baker, M. Watson, R. Alderman, J. Bannister, O. E. Gaggiotti, D. R. Gröcke, N. Patenaude, R. Harcourt

Scientific Reports 5: Article number: 16182; 10.1038/srep16182published online: 11092015; updated: 03042016

This Article contains errors. In the Methods section,

“Small samples of skin were collected from southern right whales across New Zealand and Australia using a stainless-steel biopsy dart fired from a modified veterinary capture rifle^31^ or deployed from a crossbow^32^. Samples were collected from Bremer Bay/Doubtful Island Bay, Western Australia in 1994 and 1995 as previously described^23^, and Cape Jervis/Encounter Bay, South Australia, Warrnambool and Port Fairy, Victoria, along the coast of Queensland, New South Wales, and south east Tasmania between 2001 and 2013. In New Zealand, samples were collected from southern right whales around the NZ sub-Antarctic (2006–2009) and mainland New Zealand (2003–2010) wintering grounds as previously described^24^ and a subset (n = 51) were used here as a comparison. Sampling was carried out following protocols that were approved by the Macquarie University Animal Ethics Committee, Department of Primary Industries Parks Water and Environment Animal Ethics Committee and the University of Auckland Animal Ethics Committee. Sampling was conducted in accordance with and under permits from: Department of Environment, (Australia); Department of Environment and Heritage (South Australia); Department of Primary Industries, Parks, Water and Environment (Tasmania); Department of Natural Resources and Environment (Victoria); Office of Environment and Heritage (New South Wales); Department of Conservation and Land Management (Western Australia) and the New Zealand Department of Conservation (New Zealand).”

should read:

“Small samples of skin were collected from southern right whales across New Zealand and Australia using a stainless-steel biopsy dart fired from a modified veterinary capture rifle^31^ or deployed from a crossbow^32^, and in one case, a southern right whale carcass. Samples were collected from Bremer Bay/Doubtful Island Bay, Western Australia in 1994 and 1995 as previously described^23^, and Cape Jervis/Encounter Bay, South Australia, Warrnambool and Port Fairy, Victoria, along the coast of New South Wales and south east Tasmania between 2001 and 2013. One sample was also collected in Queensland in 2014 from a southern right whale carcass, presumed killed due to ship strike. In New Zealand, samples were collected from southern right whales around the New Zealand sub-Antarctic (2006-2009) and mainland New Zealand (2003–2010) wintering grounds as previously described^24^ and a subset (n=51) were used here as a comparison. Sampling was carried out following protocols that were approved by the Macquarie University Animal Ethics Committee, Department of Primary Industries Parks Water and Environment Animal Ethics Committee and the University of Auckland Animal Ethics Committee. Sampling was conducted in accordance with and under permits from: Department of Environment, (Australia); Department of Environment and Heritage Protection (Queensland), Department of Environment and Heritage (South Australia); Department of Primary Industries, Parks, Water and Environment (Tasmania); Department of Natural Resources and Environment (Victoria); Office of Environment and Heritage (New South Wales); Department of Conservation and Land Management (Western Australia) and the New Zealand Department of Conservation (New Zealand).”

In the Acknowledgments section,

“Samples were collected in Australia under permits from: Dept. of Environment, (Australia); Dept. of Environment and Heritage (SA); Dept. of Primary Industries, Parks, Water and Environment (Tasmania); Dept. of Natural Resources and Environment (Victoria); Office of Environment and heritage (NSW); Dept. of Conservation and Land Management (WA) and Macquarie University Animal Ethics Committee. Samples were collected in New Zealand under permit from Dept. of Conservation (DOC NZ) and a protocol approved by the University of Auckland Animal Ethics Committee. Thanks to S. Childerhouse and G. Dunshea for assistance with Auckland Islands field trips and L. Boren, D. Engelhaupt and DOC NZ regional for samples collected around mainland New Zealand. Thanks to E. McClymont, D. Steel, A. Alexander and A. Sremba for help with laboratory work.”

should read:

“Samples were collected in Australia under permits from: Dept. of Environment, (Australia); Dept. of Environment and Heritage Protection (QLD) sighting ID 49787, Dept. of Environment and Heritage (SA); Dept. of Primary Industries, Parks, Water and Environment (TAS); Dept. of Natural Resources and Environment (VIC); Office of Environment and heritage (NSW); Dept. of Conservation and Land Management (WA) and Macquarie University Animal Ethics Committee. Samples were collected in New Zealand under permit from Dept. of Conservation (DOC NZ) and a protocol approved by the University of Auckland Animal Ethics Committee. Thanks to S. Childerhouse and G. Dunshea for assistance with Auckland Islands field trips, J. Meagher for sample collection in Queensland and L. Boren, D. Engelhaupt and DOC NZ regional for samples collected around mainland New Zealand. Thanks to E. McClymont, D. Steel, A. Alexander and A. Sremba for help with laboratory work.”

