# Supplementary material for: Cultural traditions across a migratory network shape the genetic structure of southern right whales around Australia and New Zealand
Source: Sci Rep. 2015 Nov 9;5:16182. doi: 10.1038/srep16182 (PMC4637828; doi:10.1038/srep16182)
Supplement: Supplementary Information [file srep16182-s1.doc]

**Cultural traditions across a migratory network shape the genetic structure of southern right whales around Australia and New Zealand**

E. L. Carroll^1,2^*, C. S. Baker^3,4^, M. Watson^5^, R. Alderman^6^, J. Bannister^7^, O. E. Gaggiotti^1^, D. R. Gröcke^8^, N. Patenaude^2,9^ and R. Harcourt^2^

1 Scottish Oceans Institute, University of St Andrews, St Andrews, Fife, KY16 8LB, Scotland

2 Department of Biological Sciences, Macquarie University, Sydney, NSW 2109, Australia

3 School of Biological Sciences, University of Auckland, Auckland 1010, New Zealand

4 Marine Mammal Institute and Department of Fisheries and Wildlife, Hatfield Marine Science Center, Oregon State University, Newport, OR 97365, USA

5 Department of the Environment, Land, Water and Planning, Barwon South West Region, Warrnambool, VIC 3280, Australia

6 Department of Primary Industries, Parks, Water and Environment, Hobart, TAS 7000, Australia

7 The Western Australian Museum, Locked Bag 49, Welshpool DC, WA 6986, Australia

8 Department of Earth Sciences, Durham University, Durham, DH1 3LE, United Kingdom

9 Collégial International Sainte-Anne, Montréal, Québec, QC H8S 2M8, Canada

*communicating author: elc6@st-andrews.ac.uk

**Supplementary Material 1: Construction of DNA and stable isotope profiles**

**DNA extraction, sex identification and mtDNA haplotype sequencing**

Total genomic DNA was extracted from skin biopsy samples using standard proteinase K digestion and phenol/chloroform methods^1^ as modified by Baker et al.^2^ for small tissue samples.

The sex of sampled whales was identified by amplification of a portion of the sex-determining region on the Y chromosome, multiplexed with an amplification of the ZFY/ZFX region as positive control^3,4^.

For Australia, we used mtDNA sequences from Carroll et al.^5^ and used the methods in this paper to generated new mitochondrial DNA control region (mtDNA) haplotypes from the samples not previously analysed. For these new sequences, we aligned and edited mtDNA sequences in either Sequencher v4.2^TM^ (Gene Codes Corporation) or Geneious v7^6^. Haplotypes were identified from a 500 bp consensus region and identified following the naming convention of Carroll et al.^5^. For New Zealand, we used 692 mtDNA haplotypes associated with unique DNA profiles generated in Carroll et al^7^.

**Microsatellite genotyping**

Seventeen microsatellite loci were amplified in individual 10 μL PCR reactions under conditions and reaction mixtures described in Supplementary Table 2. Amplicons from 4-6 loci were co-loaded for electrophoresis using an ABI3730 or an ABI3130. As a precaution against poor DNA quality, samples had to genotyped at a minimum of 10 loci to be included in subsequent analyses. Matching genotypes identified by CERVUS v3.0 were assumed to be replicate samples from the same individual and the average probability of identity (P^ID7^) was calculated from these matching genotypes. When a replicate was identified, only one copy of the genotype was retained per sampling location for subsequent analyses.

Each 96-well tray included a set of 4-7 internal controls to ensure consistent allele sizing and a negative control to detect contamination. GENEPOP v4.0^9^ was used to test for linkage disequilibrium and CERVUS v3.0 was used to test for deviations from the Hardy-Weinberg equilibrium.

**Stable isotope analysis**

A subsample of the skin biopsy sample was freeze dried and underwent lipid extraction following Todd et al^10^. Total carbon, total nitrogen content and stable isotope analysis of the approximately 500 micrograms of each sample were performed using a Costech Elemental Analyser (ECS 4010) connected to a ThermoFinnigan Delta V Advantage isotope ratio mass spectrometer. Carbon isotope ratios were corrected for 17O contribution and reported in standard delta (δ) notation in per mil (‰) relative to Vienna Pee Dee Belemnite (VPDB). Isotopic accuracy was monitored through routine analyses of in-house standards for each run, which were stringently calibrated against international standards (e.g., USGS 40, USGS 24, IAEA 600, IAEA N1, IAEA N2): this provided a linear range in δ13C between –46.7 ‰ and +2.9 ‰ and in δ15N between –4.5 ‰ and +20.4 ‰. All isotopic results are corrected against this linear range of standards. Analytical uncertainty in carbon isotope analysis was typically ±0.1 ‰ for replicate analyses of the international standards and typically <0.2 ‰ on replicate sample analysis. Analytical uncertainty in nitrogen isotope analysis was typically ±0.1 ‰ for replicate analyses of the international standards and typically <0.2 ‰ on replicate sample analysis. Total carbon and nitrogen data was obtained as part of the isotopic analysis using an internal standard (Glutamic Acid, 40.82 % C, 9.52 % N). CN ratios are expressed atomically. Isotope ratios are expressed as δ^13^C or δ ^15^N (‰) = [(*R_sample_*/*R_standard_*)-1] x 1000, where *R* is ^13^C/^12^C or ^15^N/^14^N, for δ^13^C or δ^15^N, respectively.

**Supplementary Material 2: Results from comparison of genetic data and δ^15^N values**

An interaction was indicated by plotting the mean of δ^15^N against two-way combinations of factors, so we considered GLMs with interaction terms.

For the δ^15^N data, there were four models closely ranked using ΔAIC (Supplementary Table 4). The best model indicated by residual deviance was also mtDNA*sex*state. However, models considering sex and state only, ranked highly using ΔAIC, whereas the model that only considered mtDNA was ranked last, indicating it was a weaker explanatory factor in the δ^15^N dataset. Visual inspection of the residuals of the mtDNA*sex*state models for both datasets indicated the data were normally distributed.

The randomisation test of Valenzuela et al.^11^ indicated no relationship between δ^15^N isotopic similarity and matriline (p>0.05). In addition, there was no significant relationship between the relatedness estimates of Ritland^12^ (p>0.05) and Lynch and Ritland^13^ (p>0.05) and the Euclidean distances of δ^15^N based on the results of the mantel tests.

Supplementary Table 1: Details of samples used in the analysis, including sex, sampling location (State; New South Wales: NSW, Queensland: QLD, South Australia: SA, Victoria: VIC and Western Australia: WA), mitochondrial control region haplotype (mtDNA), and the habitat classification of the sampling area as a migratory (MIG) or calving ground (CAL).

| Sample | Sex | Location | State | mtDNA | Hab |
| --- | --- | --- | --- | --- | --- |
| Eau08EA01 | F | Sydney | NSW | BakHapB+ | MIG |
| Eau10EA03 | M | Narooma | NSW | BakHapA | MIG |
| Eau10EA04 | M | Narooma | NSW | BakHapE | MIG |
| Eau10EA05 | F | Narooma | NSW | BakHapC | MIG |
| Eau10EA06 | F | Narooma | NSW | BakHapA | MIG |
| SRWEA0305 | F | Newcastle | NSW | BakHapB+ | MIG |
| SRWEA0306 | F | Merimbula | NSW | PatHap17 | MIG |
| MQ38 | F | Jervis Bay | NSW | BakHapC | MIG |
| MQ13 | M | Narooma | NSW | BakHapA | MIG |
| MQ39 | F | Jervis Bay | NSW | BakHapA | MIG |
| MQ37 | F | ? | NSW | BakHapA | MIG |
| MQ27 | F | Sunshine Coast | QLD | BakHapB+ | MIG |
| Eau0102 | F | Encounter Bay | SA | BakHapA | MIG |
| Eau0103 | M | Encounter Bay | SA | BakHapC | MIG |
| Eau0104 | M | Encounter Bay | SA | BakHapA | MIG |
| Eau0105 | F | Cape Jervis | SA | BakHapC | MIG |
| Eau0107 | F | Encounter Bay | SA | BakHapA | MIG |
| Eau0108 | M | Encounter Bay | SA | BakHapC | MIG |
| Eau0109 | M | Encounter Bay | SA | BakHapA | MIG |
| Eau0110 | M | Encounter Bay | SA | BakHapA | MIG |
| Eau0111 | M | Encounter Bay | SA | BakHapC | MIG |
| Eau0112 | F | Encounter Bay | SA | BakHapE | MIG |
| Eau0116 | M | Encounter Bay | SA | BakHapA | MIG |
| Eau0117 | M | Encounter Bay | SA | BakHapA | MIG |
| Eau0118 | F | Encounter Bay | SA | BakHapA | MIG |
| Eau0201 | F | Encounter Bay | SA | BakHapD | MIG |
| Eau0202 | F | Encounter Bay | SA | BakHapD | MIG |
| Eau0203 | F | Encounter Bay | SA | BakHapE | MIG |
| Eau0204 | F | Encounter Bay | SA | SWPJ | MIG |
| Eau0206 | M | Encounter Bay | SA | BakHapC | MIG |
| Eau0207 | M | Encounter Bay | SA | BakHapC | MIG |
| SRWEA0301 | F | Encounter Bay | SA | BakHapB+ | MIG |
| Sample | Sex | Location | State | mtDNA | Hab |
| SRWEA0302 | F | Encounter Bay | SA | BakHapB+ | MIG |
| SRWEA0303 | M | Encounter Bay | SA | BakHapC | MIG |
| Eau07Tas01 | M | Goats Beach, Storm Bay | TAS | CarHapJ | MIG |
| Eau07Tas02 | F | Goats Beach, Storm Bay | TAS | PatHap4 | MIG |
| Eau07Tas03 | M | Goats Beach, Storm Bay | TAS | BakHapA | MIG |
| Eau12Tas01 | M | Dodges Ferry | TAS | BakHapE | MIG |
| Eau12Tas02 | M | Seven Mile Beach | TAS | BakHapA | MIG |
| Eau12Tas03 | F | Cremorne | TAS | BakHapB+ | MIG |
| Eau12Tas04 | M | Adventure Bay | TAS | BakHapA | MIG |
| Eau12Tas05 | F | Adventure Bay | TAS | BakHapA | MIG |
| Eau12Tas06 | M | Adventure Bay | TAS | BakHapA | MIG |
| Eau12Tas07 | F | Adventure Bay | TAS | BakHapC | MIG |
| Eau0113 | F | Warrnambool | VIC | BakHapA | CAL |
| Eau0115 | F | Warrnambool | VIC | BakHapB+ | CAL |
| Eau0208 | F | Warrnambool | VIC | BakHapD | CAL |
| Eau0209 | M | Warrnambool | VIC | BakHapB+ | CAL |
| Eau0210 | F | Warrnambool | VIC | BakHapE | CAL |
| Eau09EA01 | F | Warrnambool | VIC | BakHapB+ | CAL |
| Eau09EA02 | F | Warrnambool | VIC | BakHapB+ | CAL |
| SRWEA0304 | F | Warrnambool | VIC | BakHapD | CAL |
| SRWEA0401 | F | Warrnambool | VIC | BakHapA | CAL |
| SRWEA0402 | F | Warrnambool | VIC | BakHapD | CAL |
| MQ29 | M | Warrnambool | VIC | BakHapC | CAL |
| MQ32 | M | Port Fairy | VIC | BakHapE | MIG |
| MQ35 | F | Port Fairy | VIC | PatHap17 | MIG |
| MQ33 | F | Port Fairy | VIC | BakHapA | MIG |
| MQ36 | F | Warrnambool | VIC | BakHapA | CAL |
| MQ34 | M | Port Fairy | VIC | BakHapC | MIG |
| WARW9502 | F | Bremer/Doubtful Is. Bay | WA | BakHapA | CAL |
| WARW9503 | M | Bremer/Doubtful Is. Bay | WA | BakHapC | CAL |
| WARW9511 | M | Bremer/Doubtful Is. Bay | WA | BakHapA | CAL |
| WARW9512 | M | Bremer/Doubtful Is. Bay | WA | BakHapC | CAL |
| Eau94WA01 | N/A | Bremer/Doubtful Is. Bay | WA | SWPJ | CAL |
| WARW9507 | F | Bremer/Doubtful Is. Bay | WA | BakHapA | CAL |
| WARW9508 | F | Bremer/Doubtful Is. Bay | WA | BakHapC | CAL |
| WARW9509 | M | Bremer/Doubtful Is. Bay | WA | BakHapE | CAL |
| WARW9510 | M | Bremer/Doubtful Is. Bay | WA | BakHapC | CAL |
| WARW9516 | M | Bremer/Doubtful Is. Bay | WA | BakHapE | CAL |
| Eau94WA02 | M | Bremer/Doubtful Is. Bay | WA | BakHapA | CAL |
| Eau94WA03 | M | Bremer/Doubtful Is. Bay | WA | BakHapA | CAL |
| Eau94WA05 | N/A | Bremer/Doubtful Is. Bay | WA | N/A | CAL |
| WARW9505 | M | Bremer/Doubtful Is. Bay | WA | BakHapF | CAL |
| Sample | Sex | Location | State | mtDNA | Hab |
| WARW9514 | F | Bremer/Doubtful Is. Bay | WA | BakHapA | CAL |
| WARW9515 | F | Bremer/Doubtful Is. Bay | WA | BakHapA | CAL |
| WARW9517 | M | Bremer/Doubtful Is. Bay | WA | BakHapA | CAL |

Supplementary Table 2: Seventeen loci used for microsatellite genotyping of southern right whales. Details include primer sequences, fluorescent label, annealing temperature (TA), magnesium concentration (mM Mg) and reference.

| **Locus** | **Primers** | **Label** | **TA (ºC)†** | **mM Mg** | **Reference** |
| --- | --- | --- | --- | --- | --- |
| **CA232** | F: CACTCAGATTAAGACTTCAGA | FAM | 55 | 2.5 | Bérubé et al.^14^ |
|  | R: GATCACATAATCTTGATCAGA |  |  |  |  |
| **EV1*Pm*** | F: CCCTGCTCCCCATTCTC | NED | 60 | 2.5 | Valsecchi & Amos |
|  | R: ATAAACTCTAATACACITCCTCCAAC |  |  |  |  |
| **EV37*Mn*** | F: AGCTTGATTTGGAAGTCATGA | NED | 54 | 2.5 | Valsecchi & Amos |
|  | R: TAGTAGAGCCGTGATAAAGTGC |  |  |  |  |
| **EV14*Pm*** | F: TAAACATCAAAGCAGACCCC | VIC | 51 | 2.5 | Valsecchi & Amos |
|  | R: CCAGAGCCAAGGTCAAGAG |  |  |  |  |
| **EV94** | F: atcctattggtccttttctgc | FAM | 55 | 2.0 | Valsecchi & Amos |
|  | R: aatagatagtgatgatgarcacacc |  |  |  |  |
| **GATA028** | F: AAAGACTGAGATCTATAGTTA | NED | 50 | 2.5 | Palsbøll et al.^16^ |
|  | R: CGCTGATAGATTAGTCTAGG |  |  |  |  |
| **GATA098** | F: TGTACCCTGGATGGATAGATT | VIC | 50 | 2.5 | Palsbøll et al.^16^ |
|  | R: ATGTCTCTCTCACACCTCACC |  |  |  |  |
| **GT23** | F: GTTCCCAGGCTCTGCACTCTG | VIC | 58 | 2.0 | Bérubé et al.^17^ |
|  | R: CATTTCCTACCCACCTGTCAT |  |  |  |  |
| **GT310** | F: gaatactcccagtagtttctc | NED | 59 | 2.0 | Bérubé et al.^17^ |
|  | R: taacttgtggaagatgccaac |  |  |  |  |
| **RW18** | F: AGAGGGAAGCAAACTGGA | FAM | 60 | 2.5 | Waldick et al.^18^ |
|  | R: GAAGGNTGCCAGACACCC |  |  |  |  |
| **RW31** | F: TATTCATGGAGTGCTTTGG | FAM | 54 | 2.0 | Waldick et al.^18^ |
|  | R: CCTAGAGTCCAGTGTGGTA |  |  |  |  |
| **RW48** | F: CCAATGACTTTTCCCTGTA | NED | 50 | 2.5 | Waldick et al.^18^ |
|  | R: GATACCGCAGTGTGTCCTG | | |  |  |
| **RW410** | F: ATGGCATTACTTCATTCTTT | VIC | 50 | 2.5 | Waldick et al.^18^ |
|  | R: GCCAAACTTACCAAATTGTG | | |  |  |
| **RW26** | F: GTCCATCCATATTACTGC | NED | 50 | 2.5 | Waldick et al.^18^ |
|  | R: CAGTTATACCTCAATGAAGC | | |  |  |
| **TR3F4** | F: TGCTCTGCAACAAGAGAAGC | FAM | 59 | 2.0 | Frasier et al.^19^ |
|  | R: GCCAAGGTTTTAGAGAGAGTG | | |  |  |
| **TR3G1** | F: CTCCGCAACAAGAGAGGC | FAM | *A | 2.5 | Frasier et al.^19^ |
|  | R: CTTCCTGGGTACAAGCCC | | |  |  |
| **TR3G2** | F: CTGCGGTGTTGGTTAATAGC | VIC | 50 | 2.5 | Frasier et al.^19^ |
|  | R: CCTGACATTTTCTGTGTCCC | | |  |  |

**†**These reactions have cycling conditions of (i) an initial denaturing step at 94 °C for 3 min; (ii) 35 cycles at 94 °C for 30 sec, TA for 30 sec and 72 °C for 30 sec; and (iii) a final extension step at 72 °C for 10 min. *A indicates this primer pair had a touchdown PCR protocol: for the cycling, each annealing temperature is used for five cycles before stepping down to the next annealing temperature; the final annealing temperature is used for 10 cycles, resulting in a total of 30 cycles. Annealing temperatures are 68 °C, 64 °C, 61 °C, 58 °C and 55 °C.

Supplementary Table 3: DNA profiles for southern right whales recaptured between days or years on the Australian wintering grounds. DNA profiles comprise genetically identified sex (Sex), mitochondrial control region haplotype (mtDNA: haplotype code as described in Carroll *et al.* 2011*b*) and multilocus genotype (EV1 – TR3G2). The sampling location (STATE), probability of identity (P_ID_), number of matching loci (N_M_), and number of loci each sample is amplified at (N_loci_) are also reported. Missing data at a locus are denoted by 0. Most notable matches are of two females that were sampled in different years in Warrnambool, Victoria. The first female^A^ was captured in 2001 and recaptured in 2009 (as previously reported in Carroll et al 2011), and the second female^B^ was captured in 2004 and subsequently recaptured in 2009. In addition, another female^C^ was sampled in 2007 in two different locations along the NSW coast – Merimbula and Eden.

| **Sample** | **Year** | **N_M_/P_ID_** | **mtDNA** | **sex** | **STATE** | **CA232** | | **EV1** | | **EV14** | | **EV37** | | **EV94** | | **GATA28** | | **GATA98** | |
| --- | --- | --- | --- | --- | --- | --- | --- | --- | --- | --- | --- | --- | --- | --- | --- | --- | --- | --- | --- |
| Eau01EA03 | 2001 | 13/  1.94E-17 | BakHapC | M | SA | 136 | 136 | 136 | 144 | 133 | 133 | 195 | 203 | 200 | 200 | 178 | 178 | 112 | 112 |
| Eau01EA06 | 2001 |  | BakHapC | M | SA | 0 | 0 | 136 | 144 | 133 | 133 | 195 | 203 | 0 | 0 | 178 | 178 | 112 | 112 |
|  |  |  |  |  |  |  |  |  |  |  |  |  |  |  |  |  |  |  |  |
| Eau02EA04 | 2002 | 11/ | SWPJ | F | SA | 142 | 150 | 122 | 144 | 133 | 141 | 203 | 203 | 196 | 200 | 166 | 166 | 112 | 112 |
| Eau02EA05 | 2002 | 1.18E-16 | SWPJ | F | SA | 0 | 0 | 122 | 144 | 133 | 141 | 203 | 203 | 0 | 0 | 166 | 166 | 112 | 112 |
|  |  |  |  |  |  |  |  |  |  |  |  |  |  |  |  |  |  |  |  |
| MQ26^C^ | 2007 | 11/ | CarHapJ | F | NSW | 0 | 0 | 122 | 136 | 120 | 133 | 199 | 205 | 0 | 0 | 174 | 178 | 112 | 116 |
| Eau07EA01 | 2007 | 11 | CarHapJ | F | NSW | 0 | 0 | 122 | 136 | 120 | 133 | 199 | 205 | 0 | 0 | 174 | 178 | 112 | 116 |
|  |  |  |  |  |  |  |  |  |  |  |  |  |  |  |  |  |  |  |  |
| **Sample** | **Year** | **N_M_/P_ID_** | **mtDNA** | **sex** | **STATE** | **CA232** | | **EV1** | | **EV14** | | **EV37** | | **EV94** | | **GATA28** | | **GATA98** | |
| Eau10EA02 | 2010 | 13/ | BakHapB+ | F | NSW | 136 | 136 | 134 | 134 | 122 | 133 | 189 | 199 | 0 | 0 | 166 | 166 | 116 | 120 |
| Eau10EA05 | 2010 | 7.78E-20 | BakHapB+ | F | NSW | 0 | 0 | 134 | 134 | 122 | 133 | 189 | 199 | 0 | 0 | 166 | 166 | 116 | 120 |
|  |  |  |  |  |  |  |  |  |  |  |  |  |  |  |  |  |  |  |  |
| Eau01EA14^A^ | 2001 | 13/ | BakHapB+ | F | VIC | 140 | 140 | 142 | 148 | 133 | 141 | 193 | 203 | 0 | 0 | 168 | 178 | 112 | 116 |
| Eau09EA02 | 2009 | 2.48E-18 | BakHapB+ | F | VIC | 0 | 0 | 142 | 148 | 133 | 141 | 193 | 203 | 0 | 0 | 168 | 178 | 112 | 116 |
|  |  |  |  |  |  |  |  |  |  |  |  |  |  |  |  |  |  |  |  |
| Eau04EA01^B^ | 2004 | 9/ | BakHapA | F | VIC | 140 | 140 | 130 | 138 | 131 | 141 | 193 | 199 | 196 | 200 | 166 | 170 | 104 | 116 |
| MQ28 | 2009 | 9.31E-14 | BakHapA | F | VIC | 0 | 0 | 130 | 138 | 131 | 141 | 193 | 199 | 0 | 0 |  |  | 104 | 116 |
|  |  |  |  |  |  |  |  |  |  |  |  |  |  |  |  |  |  |  |  |
| MQ31 | 2012 | 11/ | BakHapE | M | VIC | 0 | 0 | 126 | 148 | 133 | 135 | 189 | 203 | 0 | 0 | 166 | 178 | 116 | 116 |
| MQ32 | 2012 | 1.04E-13 | BakHapE | M | VIC | 0 | 0 | 126 | 148 | 133 | 135 | 189 | 203 | 0 | 0 | 166 | 178 | 116 | 116 |

| **Sample** | **GT23** | | **GT12** | | **GT310** | | **RW18** | | **RW31** | | **RW410** | | **RW48** | | **TR3F4** | | **TR3G1** | | **TR3G2** | | | **N_loci_** | |
| --- | --- | --- | --- | --- | --- | --- | --- | --- | --- | --- | --- | --- | --- | --- | --- | --- | --- | --- | --- | --- | --- | --- | --- |
| Eau01EA03 | 112 | 120 | 138 | 138 | 0 | 0 | 199 | 199 | 117 | 125 | 122 | 124 | 203 | 203 | 301 | 333 | 238 | 238 | 176 | | 184 | 16 | |
| Eau01EA06 | 112 | 120 | 0 | 0 | 0 | 0 | 199 | 199 | 117 | 125 | 122 | 124 | 203 | 203 | 301 | 333 | 238 | 238 | 176 | | 184 | 13 | |
|  |  |  |  |  |  |  |  |  |  |  |  |  |  |  |  |  |  |  |  | |  |  | |
| Eau02EA04 | 118 | 120 | 138 | 138 | 0 | 0 | 187 | 193 | 121 | 123 | 108 | 122 | 197 | 205 | 305 | 345 | 214 | 226 | 184 | | 188 | 16 | |
| Eau02EA05 | 0 | 0 | 0 | 0 | 0 | 0 | 0 | 0 | 121 | 123 | 108 | 122 | 197 | 205 | 305 | 345 | 214 | 226 | 184 | | 188 | 11 | |
|  |  |  |  |  |  |  |  |  |  |  |  |  |  |  |  |  |  |  |  | |  |  | |
| MQ26 | 114 | 116 | 0 | 0 | 0 | 0 | 0 | 0 | 123 | 127 | 118 | 122 | 197 | 205 | 301 | 333 | 206 | 226 | 0 | | 0 | 11 | |
| Eau07EA01 | 114 | 116 | 0 | 0 | 0 | 0 | 193 | 199 | 123 | 127 | 118 | 122 | 197 | 205 | 301 | 333 | 206 | 226 | 176 | | 180 | 13 | |
|  |  |  |  |  |  |  |  |  |  |  |  |  |  |  |  |  |  |  |  | |  |  | |
| Eau10EA02 | 112 | 116 | 0 | 0 | 98 | 100 | 195 | 225 | 123 | 125 | 118 | 120 | 211 | 211 | 329 | 333 | 210 | 210 | 176 | | 176 | 15 | |
| Eau10EA05 | 112 | 116 | 0 | 0 | 0 | 0 | 195 | 225 | 123 | 125 | 118 | 120 | 211 | 211 | 329 | 333 | 210 | 210 | 176 | | 176 | 13 | |
|  |  |  |  |  |  |  |  |  |  |  |  |  |  |  |  |  |  |  |  | |  |  | |
| **Sample** | **GT23** | | **GT122** | | **GT310** | | **RW18** | | **RW31** | | **RW410** | | **RW48** | | **TR3F4** | | **TR3G1** | | | **TR3G2** | | | **N_loci_** |
| Eau01EA14 | 114 | 120 | 0 | 0 | 98 | 102 | 187 | 193 | 125 | 125 | 108 | 122 | 197 | 205 | 309 | 317 | 210 | 218 | 172 | | 184 | 15 | |
| Eau09EA02 | 114 | 120 | 0 | 0 | 0 | 0 | 187 | 193 | 125 | 125 | 108 | 122 | 197 | 205 | 309 | 317 | 210 | 218 | 172 | | 184 | 13 | |
|  |  |  |  |  |  |  |  |  |  |  |  |  |  |  |  |  |  |  |  | |  |  | |
| Eau04EA01 | 112 | 114 | 138 | 138 | 0 | 0 | 0 | 0 | 125 | 125 | 124 | 124 | 209 | 211 | 301 | 305 | 238 | 238 | 0 | | 0 | 14 | |
| MQ28 | 112 | 114 | 0 | 0 | 0 | 0 | 195 | 213 | 125 | 125 | 124 | 124 | 209 | 211 | 301 | 305 | 238 | 242 | 176 | | 180 | 12 | |
|  |  |  |  |  |  |  |  |  |  |  |  |  |  |  |  |  |  |  |  | |  |  | |
| MQ31 | 120 | 120 | 0 | 0 | 0 | 0 | 0 | 0 | 121 | 125 | 120 | 124 | 197 | 211 | 301 | 337 | 210 | 238 | 0 | | 0 | 11 | |
| MQ32 | 120 | 120 | 0 | 0 | 0 | 0 | 0 | 0 | 121 | 125 | 120 | 124 | 197 | 211 | 301 | 337 | 210 | 238 | 0 | | 0 | 11 | |
|  |  |  |  |  |  |  |  |  |  |  |  |  |  |  |  |  |  |  |  | |  |  | |

Supplementary Table 4: Estimated levels of genetic diversity of southern right whale sampling locations and stocks. The sample size (Nm), number of mitochondrial DNA (mtDNA) haplotypes (N_H_), haplotype (*h*) and nucleotide diversity (π) are estimated based on 500 bp of the mtDNA control region. The microsatellite sample size (2N), average number of alleles (k) and observed (H_OBS_) and expected heterozygosities (H_EXP_) are based on 17 microsatellite loci.

|  |  | mtDNA | | |  | microsatellite | | |
| --- | --- | --- | --- | --- | --- | --- | --- | --- |
| Region | Nm | N_H_ | *h*±SD | π±SD | 2N | k | H_OBS_ | H_EXP_ |
| NSW | 12 | 6 | 0.82±0.96 | 1.41±0.80 | 24 | 6.41 | 0.77 | 0.77 |
| TAS | 10 | 6 | 0.78±0.14 | 1.16±0.68 | 20 | 6.11 | 0.75 | 0.77 |
| VIC | 16 | 6 | 0.86±0.05 | 1.99±1.08 | 32 | 6.65 | 0.81 | 0.79 |
| SA | 22 | 6 | 0.77±0.06 | 1.67±0.90 | 44 | 7.12 | 0.70 | 0.74 |
| WA | 16 | 5 | 0.71±0.09 | 1.51±0.83 | 34 | 6.47 | 0.78 | 0.76 |
| Stocks |  |  |  |  |  |  |  |  |
| SEA | 39 | 8 | 0.81±0.04 | 1.59±0.84 | 78 | 8.47 | 0.77 | 0.77 |
| SWA | 38 | 7 | 0.74±0.50 | 1.58±0.83 | 78 | 7.94 | 0.75 | 0.76 |
| NZ | 692 | 12 | 0.76±0.01 | 1.39±0.07 | 102 | 8.65 | 0.71 | 0.75 |

Supplementary Table 5: Mitochondrial control region haplotype frequencies across southern right whale calving grounds (New Zealand (NZ), Victoria (VIC) and Western Australia (WA)) and migratory corridors (New South Wales (NSW), Tasmania (TAS), Victoria (VIC-M) and South Australia (SA)).

|  | NZ-C | NSW-M | VIC-C | VIC-M | TAS | SA | WA |
| --- | --- | --- | --- | --- | --- | --- | --- |
| BakHapA | 231 | 5 | 3 | 1 | 5 | 8 | 8 |
| BakHapB’ | 87 | 0 | 0 | 0 | 0 | 0 | 0 |
| BakHapB+ | 207 | 2 | 4 | 0 | 1 | 2 | 0 |
| BakHapC | 58 | 2 | 1 | 1 | 1 | 7 | 4 |
| BakHapD | 82 | 0 | 3 | 0 | 0 | 2 | 0 |
| BakHapE | 6 | 1 | 1 | 1 | 1 | 2 | 2 |
| BakHapF | 0 | 0 | 0 | 0 | 0 | 0 | 1 |
| CarHapJ | 1 | 1 | 0 | 0 | 1 | 0 | 0 |
| CarHapK | 2 | 0 | 0 | 0 | 0 | 0 | 0 |
| PatHap04.1 | 1 | 0 | 0 | 0 | 0 | 0 | 0 |
| PatHap04.2 | 12 | 0 | 0 | 0 | 1 | 0 | 0 |
| PatHap17 | 1 | 1 | 0 | 1 | 0 | 0 | 0 |
| PatMalHapB | 4 | 0 | 0 | 0 | 0 | 0 | 0 |
| SWPJ | 0 | 0 | 0 | 0 | 0 | 1 | 1 |
| Total | 692 | 12 | 12 | 4 | 10 | 22 | 16 |

Supplementary Table 6: Pairwise comparisons of southern right whale calving grounds, based on samples collected from New Zealand (NZ), Victoria (VIC) and Western Australia (WA) and migratory corridors, based on samples collected from South Australia (SA), Tasmania (TAS) and New South Wales (NSW). A. mtDNA-based F_ST_ (bottom left quadrant) and Φ_ST_ (top right quadrant) B. microsatellite-based F_ST_ (bottom left quadrant) and Jost’s D (top right quadrant)

| A. | WA | SA | VIC | TAS | NSW | NZ |  |
| --- | --- | --- | --- | --- | --- | --- | --- |
| WA |  | 0.000 | 0.057 | 0.062 | 0.006 | 0.164** |  |
| SA | 0.000 |  | 0.010 | 0.082 | 0.012 | 0.144** |  |
| VIC | 0.098* | 0.032 |  | 0.048 | 0.004 | 0.029 |  |
| TAS | 0.000 | 0.000 | 0.032 |  | 0.000 | 0.025 |  |
| NSW | 0.000 | 0.000 | 0.002 | 0.000 |  | 0.005 |  |
| NZ | 0.088** | 0.055* | 0.000 | 0.029 | 0.010 |  |  |
|  |  |  |  |  |  |  |  |
| B. | WA | SA | VIC | TAS | NSW | NZ |  |
| WA |  | 0.026 | 0.038* | 0.062** | 0.003 | 0.051** |  |
| SA | 0.005 |  | 0.000 | 0.036* | 0.028 | 0.016 |  |
| VIC | 0.011* | 0.000 |  | 0.015 | 0.046 | 0.000** |  |
| TAS | 0.018** | 0.010* | 0.004 |  | 0.054 | 0.020 |  |
| NSW | 0.002 | 0.007 | 0.014 | 0.015 |  | 0.024 |  |
| NZ | 0.016** | 0.003 | 0.000** | 0.005 | 0.006 |  |  |

*p<0.05; **p<0.01

Supplementary Table 7: Results of generalised linear modelling of δ^13^C and δ^15^N values. Mitochondrial control region haplotype (mtDNA), sex or sampling region (state; sampling location from Table 1 and Supplementary Table 3) were fitted as explanatory variables for δ^13^C or δ^15^N profiles of 66 southern right whales sampled around Australia. Interaction terms are denoted by two factors with “:” between them. Models are ranked using ΔAIC and model weight is listed.

| δ^13^C model | ΔAIC | Weight |
| --- | --- | --- |
| MTDNA + SEX + STATE + STATE:SEX | 0.00 | 0.69 |
| MTDNA + SEX + STATE + SEX:MTDNA + STATE:SEX | 2.46 | 0.20 |
| MTDNA + SEX + STATE + STATE:MTDNA + STATE:SEX | 6.27 | 0.03 |
| MTDNA + STATE | 6.85 | 0.02 |
| MTDNA + STATE + STATE:MTDNA | 7.71 | 0.01 |
| MTDNA + SEX + STATE | 8.24 | 0.01 |
| SEX + STATE + STATE:SEX | 8.57 | 0.01 |
| MTDNA + SEX + STATE + STATE:MTDNA | 9.16 | 0.01 |
| MTDNA + SEX + STATE + SEX:MTDNA + STATE:MTDNA + STATE:SEX | 9.44 | 0.01 |
| MTDNA + SEX + STATE + SEX:MTDNA | 10.44 | 0.00 |
| MTDNA | 11.33 | 0.00 |
| MTDNA + SEX | 12.57 | 0.00 |
| SEX + STATE | 13.75 | 0.00 |
| MTDNA + SEX + STATE + SEX:MTDNA + STATE:MTDNA | 14.18 | 0.00 |
| MTDNA + SEX + SEX:MTDNA | 14.27 | 0.00 |
| STATE | 14.89 | 0.00 |
| SEX | 19.93 | 0.00 |
| δ^15^N model | ΔAIC | Weight |
| MTDNA + SEX + STATE + SEX:MTDNA + STATE:MTDNA | 0.00 | 0.82 |
| MTDNA + SEX + STATE + SEX:MTDNA + STATE:MTDNA + STATE:SEX | 5.31 | 0.06 |
| MTDNA + SEX + STATE + STATE:MTDNA + STATE:SEX | 6.66 | 0.03 |
| STATE | 6.86 | 0.03 |
| SEX + STATE | 8.49 | 0.01 |
| SEX | 8.57 | 0.01 |
| SEX + STATE + STATE:SEX | 9.39 | 0.01 |
| MTDNA + SEX + STATE + SEX:MTDNA | 10.60 | 0.00 |
| MTDNA + SEX + STATE + SEX:MTDNA + STATE:SEX | 11.77 | 0.00 |
| MTDNA + SEX + SEX:MTDNA | 12.63 | 0.00 |
| MTDNA + STATE + STATE:MTDNA | 14.60 | 0.00 |
| MTDNA + SEX + STATE + STATE:MTDNA | 16.58 | 0.00 |
| MTDNA | 17.06 | 0.00 |
| MTDNA + STATE | 18.01 | 0.00 |
| MTDNA + SEX | 19.06 | 0.00 |
| MTDNA + SEX + STATE | 19.56 | 0.00 |
| MTDNA + SEX + STATE + STATE:SEX | 20.28 | 0.00 |

Supplementary Table 8: Pairwise estimates of migration between southern right whale calving grounds derived from the Lamarc analysis of mtDNA control regions. Calving grounds are New Zealand (NZ), Victoria (VIC) and Western Australia (WA).

|  |  | Migration to | | |
| --- | --- | --- | --- | --- |
|  |  | NZ | VIC | WA |
| Migration  from | NZ |  | 65.7 (17.6-100) | 52.7 (5.9-100) |
|  | VIC | 69.8 (26-100) |  | 65.4 (19.2-100) |
|  | WA | 53.9 (6.4-100) | 58.0 (8.7-100) |  |

Supplementary Table 9: Estimates of θ for southern right whale calving grounds in Western Australia (WA), Victoria (VIC) and New Zealand (NZ) and the derived statistics of mean effective female population size (*N_e(f)_*) and mean census population size (*N_C_*). θ was estimated using mtDNA control region haplotypes (500 bp) and program LAMARC. The difference between results presented here and in Table 3 of the manuscript is that these results are from an analysis that explored a range of mutation rates (2x10^-8^ to 1x10^-7^), derived from ancient and contemporary bowhead whale mtDNA control region sequences^20,21^.

|  | WA | VIC | NZ |
| --- | --- | --- | --- |
| θ  (95% HPD Interval) | 4.72E-3  (8.31E-5, 0.0115) | 3.36E-3  (5.41E-5, 9.15E-3) | 5.55E-3  (3.61E-4, 0.0138) |
| *N_e(f)_*  (95% CL) | 3,989  (471, 13,615) | 2,993  (248, 11,773) | 4,892  (574,17,629) |
| *N_C_*  (95% CL) | 28,487  (3581, 108,564) | 21,786  (1823, 91,638) | 35,442  (4,042, 135,420) |

REFERENCES

1. Sambrook, J., Fritsch, E. F. & Maniatis, T. *Molecular Cloning: A Laboratory Manual 2nd ed.* (Cold Spring Harbor Laboratory Press, 1989).

2. Palumbi, S. & Baker, C. S. Contrasting population structure from nuclear intron and mtDNA of humpback whales. *Mol. Biol. Evol.* **11,** 426–435 (1994).

3. Aasen, E. & Medrano, J. Amplification of the ZFX and ZFY genes for sex identification in humans, cattle, sheep and goats. *Nat. Biotechnol.* **8,** 1279–1281 (1990).

4. Gilson, A., Syvanen, M., Levine, K. & Banks, J. Deer gender determination by polymerase chain reaction: validation study and application to tissues, bloodstains, and hair forensic samples from California. *Calif. Fish Game* **84,** 159–169 (1998).

5. Carroll, E. L. *et al.* Population structure and individual movement of southern right whales around New Zealand and Australia. *Mar. Ecol. Prog. Ser.* **432,** 257–268 (2011).

6. Kearse, M. *et al.* Geneious Basic: An integrated and extendable desktop software platform for the organization and analysis of sequence data. *Bioinformatics* **28,** 1647–1649 (2012).

7. Carroll, E. L. *et al.* Accounting for female reproductive cycles in a superpopulation capture recapture framework. *Ecol. Appl.* **23,** 1677–1690 (2013).

8. Paetkau, D. & Strobeck, C. Microsatellite analysis of genetic variation in black bear populations. *Mol. Ecol.* **3,** 489–495 (1994).

9. Rousset, F. Genepop’007: a complete re-implementation of the GENEPOP software for Windows and Linux. *Mol. Ecol. Resour.* **8,** 103–106 (2008).

10. Todd, S., Ostrom, P., Lien, J. & Abrajano, J. Use of Biopsy Samples of Humpback Whale (<i>Megaptera novaeangliae<i/>) Skin for Stable Isotope (δ 13 C) Determination. *J. Northwest Atl. Fish. Sci.* **22,** 71–76 (1997).

11. Valenzuela, L., Sironi, M., Rowntree, V. & Seger, J. Isotopic and genetic evidence for culturally inherited site fidelity to feeding grounds in southern right whales (*Eubalaena australis*). *Mol. Ecol.* **18,** 782–791 (2009).

12. Ritland, K. Estimators for pairwise relatedness and individual inbreeding coefficients. *Genet. Res.* **67,** 175–185 (1996).

13. Lynch, M. & Ritland, K. Estimation of Pairwise Relatedness With Molecular Markers. *Genetics* **152,** 1753–1766 (1999).

14. Bérubé, M. *et al.* Polymorphic microsatellite loci isolated from humpback whale, Megaptera novaeangliae and fin whale, balaenoptera physalus. *Conserv. Genet.* **6,** 631–636 (2005).

15. Valsecchi, E. & Amos, W. Microsatellite markers for the study of cetacean populations. *Mol. Ecol.* **5,** 151–156 (1996).

16. Palsbøll, P., Bérubé, M., Larsen, A. H. & Jørgensen, H. Primers for the amplification of tri- and tetramer microsatellite loci in baleen whales. *Mol. Ecol.* **6,** 893–895 (1997).

17. Bérubé, M., Jørgensen, H., McEwing, R. & Palsbøll, P. Polymorphic di-nucleotide microsatellite loci isolated from the humpback whale *Megaptera novaeangliae*. *Mol. Ecol.* **9,** 2181–2183 (2000).

18. Waldick, R. C., Brown, M. W. & White, B. N. Characterization and isolation of microsatellite loci from the endangered North Atlantic right whale. *Mol. Ecol.* **8,** 1763–1765 (1999).

19. Frasier, T. R. *et al.* Characterization of tetranucleotide microsatellite loci and development and validation of multiplex reactions for the study of right whale species (genus Eubalaena). *Mol. Ecol. Notes* **6,** 1025–1029 (2006).

20. Rooney, A., Honeycutt, R. & Derr, J. Historical population size change of bowhead whales inferred from DNA sequence polymorphism data. *Evolution (N. Y).* **55,** 1678–1685 (2001).

21. Ho, S. Y. W. *et al.* Bayesian estimation of substitution rates from ancient DNA sequences with low information content. *Syst. Biol.* **60,** 366–375 (2011).
